# Recycling Energy to Restore Impaired Ankle Function during Human Walking

**DOI:** 10.1371/journal.pone.0009307

**Published:** 2010-02-17

**Authors:** Steven H. Collins, Arthur D. Kuo

**Affiliations:** 1 Department of Biomechanical Engineering, Delft University of Technology, Delft, The Netherlands; 2 Departments of Mechanical and Biomedical Engineering, University of Michigan, Ann Arbor, Michigan, United States of America; Universidad Europea de Madrid, Spain

## Abstract

**Background:**

Humans normally dissipate significant energy during walking, largely at the transitions between steps. The ankle then acts to restore energy during push-off, which may be the reason that ankle impairment nearly always leads to poorer walking economy. The replacement of lost energy is necessary for steady gait, in which mechanical energy is constant on average, external dissipation is negligible, and no net work is performed over a stride. However, dissipation and replacement by muscles might not be necessary if energy were instead captured and reused by an assistive device.

**Methodology/Principal Findings:**

We developed a microprocessor-controlled artificial foot that captures some of the energy that is normally dissipated by the leg and “recycles” it as positive ankle work. In tests on subjects walking with an artificially-impaired ankle, a conventional prosthesis reduced ankle push-off work and increased net metabolic energy expenditure by 23% compared to normal walking. Energy recycling restored ankle push-off to normal and reduced the net metabolic energy penalty to 14%.

**Conclusions/Significance:**

These results suggest that reduced ankle push-off contributes to the increased metabolic energy expenditure accompanying ankle impairments, and demonstrate that energy recycling can be used to reduce such cost.

## Introduction

The ankle normally produces a larger burst of work than any other joint during walking [1]. Ankle impairments following amputation, joint fusion or stroke typically reduce ankle work and increase metabolic energy expenditure by at least 20% [2], comparable to carrying an extra 15 kg load [3] or walking 20% faster [4], regardless of intervention [5]–[7]. Ankle function might be restored by powering the joint directly, a technique that shows promise [8]–[10] but requires large motors and energy sources that limit range or add bulk. We propose an alternative, which is to restore ankle work simply by recycling energy that is normally dissipated as negative work.

**Figure 1:**
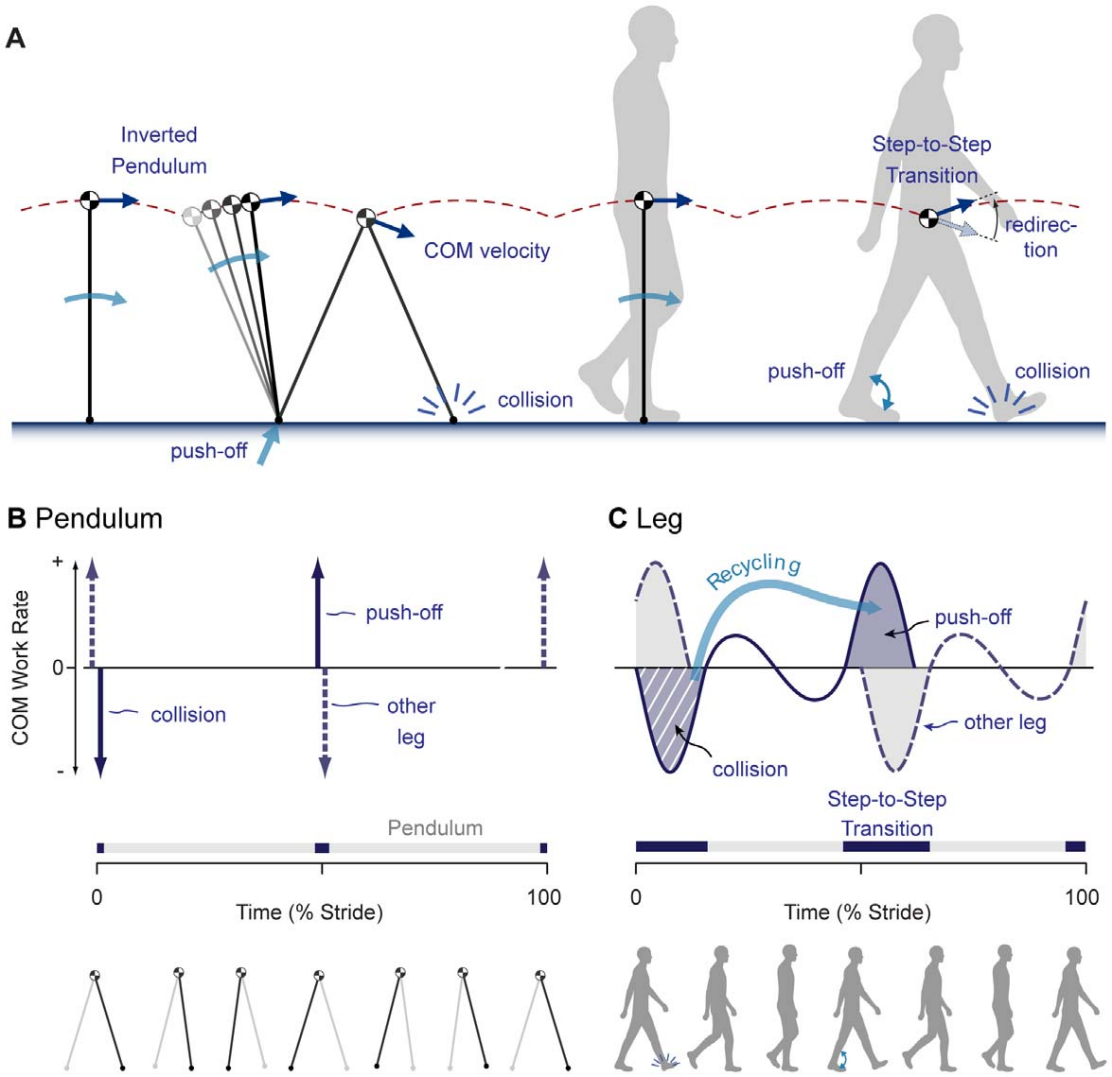
Mechanics of human walking and energy recycling. (**A**) The stance leg acts similarly to an inverted pendulum to support the body center of mass. The center of mass velocity is redirected between steps when the other leg contacts the ground with a dissipative collision. (**B**) The rate of work performed on the center of mass by ideal pendulum-like legs vs. stride time. Work is theoretically minimized by pushing off impulsively (indicated by arrows) just before the opposite leg's collision (step-to-step transition indicated by darkened intervals above time axis). (**C**) Conceptual plot of center of mass work rate for human-like legs vs. stride time. Imperfectly rigid legs will smooth out the impulses, but the collision (hatched area) is nevertheless a possible source of energy for recycling if it can be captured, stored, and later released for push-off.

Much of the dissipation in normal walking occurs when the body center of mass velocity is redirected at the transition between steps. During each step, the stance leg behaves similarly to an inverted pendulum as it transports the center of mass along an arced path (Figure 1). When the other leg contacts the ground, it flexes slightly and performs dissipative negative work as it redirects the center of mass to the arced path of the next step as part of the step-to-step transition [11], [12]. To walk at steady speed, all dissipation must be offset by an equal amount of positive work [11]–[14]. Total work may theoretically be minimized if the positive work is performed by trailing leg push-off and timed immediately before heel-strike, reducing the change in center of mass velocity performed by the collision [15]–[17]. This reduces both the dissipation and the amount of positive work needed to offset the loss. Normal ankle push-off appears appropriate for this purpose, performing positive work beginning just before and in nearly equal magnitude to the collision loss [12], [18]. If the collision energy can be successfully recycled, it may therefore be sufficient to supplement an impaired push-off. We tested this concept in controlled human experiments using an artificial foot.

## Materials and Methods

**Figure 2:**
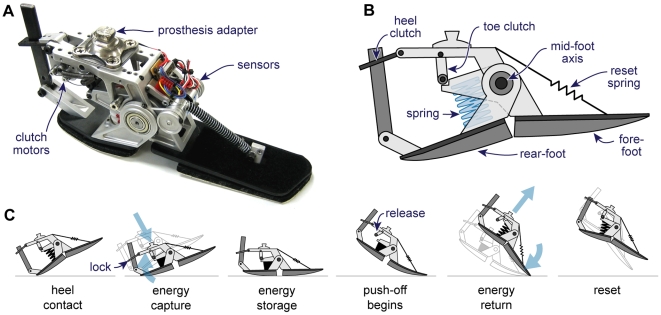
Energy recycling foot. (**A**) Prototype energy recycling device. (**B**) Schematic design showing the energy-storing spring, clutches, and independent rear- and fore-foot components. (**C**) The energy recycling sequence. Following heel-strike, the rear-foot compresses a coil spring, which is locked by a one-way clutch to capture energy. The spring remains locked until a force sensor detects loading in the fore-foot, releasing a separate clutch that allows the spring to return energy for push-off as the fore-foot is unloaded, at the beginning of push-off. The entire device resets its configuration during the swing phase.

We developed an energy-recycling artificial foot (Figure 2, Movie S1) that captures collision energy and returns it for push-off. The proof-of-concept device approximates the size and form of a conventional prosthetic foot, but has separate rear-foot and fore-foot components that rotate about a medio-lateral axis at mid-foot. When the heel contacts the ground at the beginning of a stride, the rear-foot component rotates and compresses a coil spring. At maximum compression, the rear-foot is latched by a continuous one-way clutch. Rather than releasing the spring energy spontaneously as in conventional elastic prostheses [19], [20], our device stores it until sufficient load is detected on the fore-foot. It then releases the fore-foot, and the spring provides push-off as the person begins to unload the trailing leg, with timing similar to normal ankle push-off. A small return spring resets the device during the ensuing swing phase, so that the rear-foot is in position for the next step. All of the energy capture is performed passively, so that the only active elements are a microcontroller and two micro-motors that release the energy-storing spring and reset the mechanism. The device is powered by a small battery at about 0.8 W of electricity. Active control of energy storage and return distinguishes this device from conventional prosthetic feet with passive elastic elements, which have not been found to significantly reduce the metabolic penalty of walking with ankle impairment [5]–[7], while low electrical power requirements distinguish it from other robotic prostheses [10].

We tested the artificial foot on able-bodied human subjects (N = 11, male, 19–28 yrs) walking with an artificially-immobilized ankle. Subjects wore the device (1.37 kg) on one leg using a prosthesis simulator [21], [22], a rigid boot that immobilizes the ankle and provides a prosthesis attachment beneath the foot. This allowed direct comparison between normal walking and prosthesis test conditions. Subjects also wore a lift shoe on the other foot to equalize height. The device was compared against a Conventional Prosthetic foot (Seattle LightFoot 2, Seattle Systems, Poulsbo, WA), representing a typical intervention for lower limb loss. Three conditions were applied in random order: walking with the Energy Recycling artificial foot, walking with a weight-matched Conventional Prosthesis, and Normal walking in street shoes, all at a speed of 1.25 m s^−1^. Mechanical performance was recorded through motion capture and a forceplate-instrumented treadmill [23] (Figure S1). We used motion and force data to estimate the work captured and returned by the device, the work performed by the human leg and device on the center of mass, and the work performed at each biological joint. We also recorded rates of oxygen consumption to estimate metabolic energy expenditure, reported as the net rate above that for quiet standing. Study protocol was approved by the University of Michigan Institutional Review Board, and written informed consent was obtained from all subjects after the nature and possible consequences of the study were explained. Details of these methods can be found in the supporting materials and methods section of Text S1.

## Results and Discussion

The Conventional Prosthesis reduced ankle push-off and increased metabolic expenditure for all subjects. The Energy Recycling artificial foot captured collision energy and returned it as positive ankle work later in stance, resulting in greater push-off and lower metabolic expenditure than with the Conventional Prosthesis.

**Figure 3:**
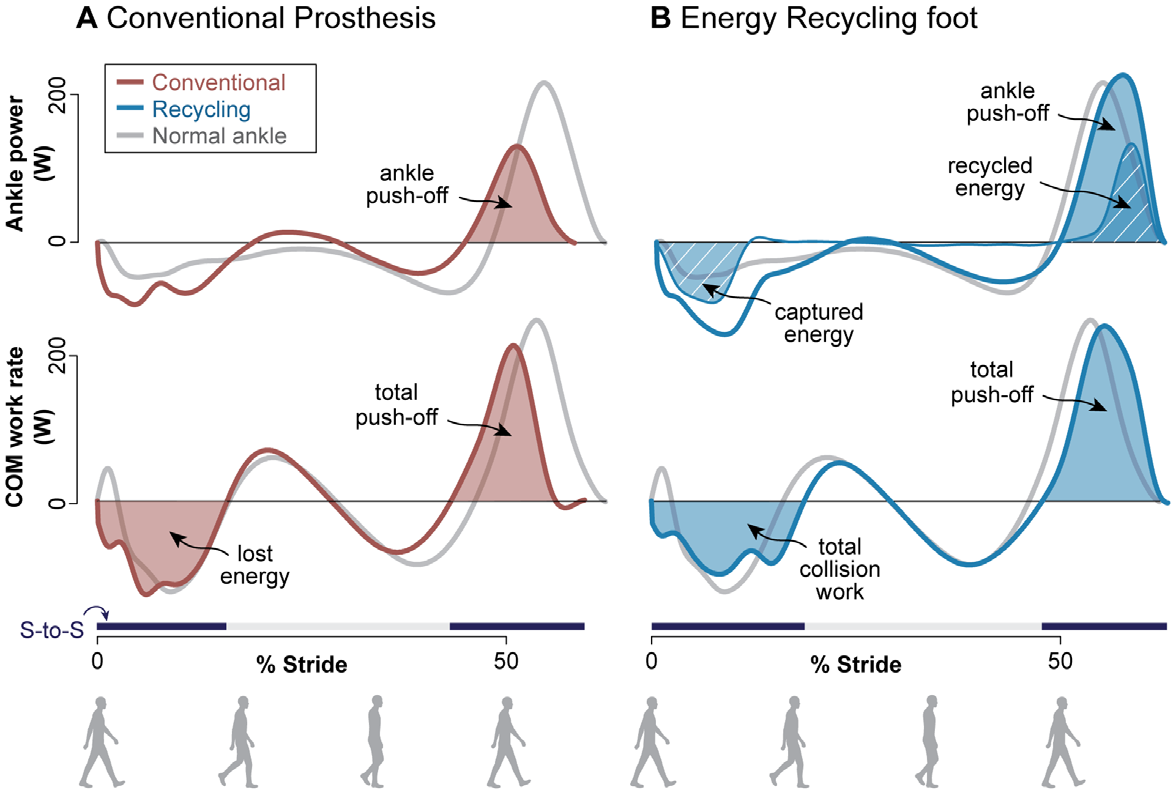
Measured work rates over a walking stride. Power produced by normal and artificial ankles (top), and rate of work performed on the center of mass by the entire leg and device (bottom), with (**A**) the Conventional Prosthetic foot and (**B**) the Energy Recycling foot. The Energy Recycling foot captured significant energy early in the stance phase (hatched area) and returned it at push-off (hatched area), resulting in greater positive ankle work than the Conventional Prosthetic foot. The center of mass work rate shows that the entire leg and device produced total push-off work closer to Normal. Although more energy was absorbed at the ankle, collision work for the entire leg and device increased little compared to Normal. Data are averaged across subjects (*n* = 11). Step-to-step transition periods are indicated by bars labeled “S-to-S” above the time axis.

**Figure 4:**
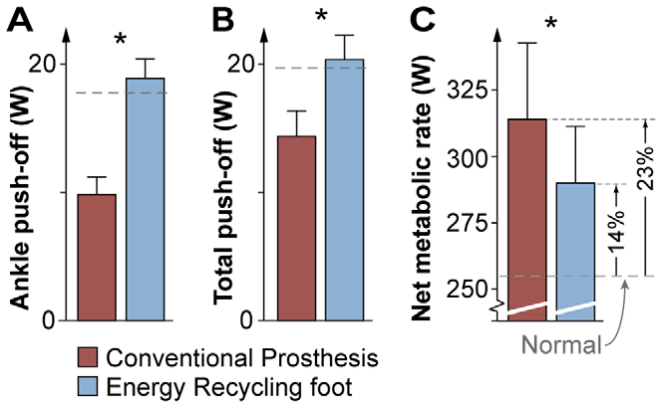
Average push-off power and net metabolic energy expenditure. (**A**) The Energy Recycling foot provided ankle push-off work at more than twice the rate of the Conventional Prosthetic foot, restoring ankle push-off to that of Normal walking (dashed line). (**B**) Subjects produced greater total push-off work with the entire leg and device on the center of mass with the Energy Recycling foot, comparable to Normal. (**C**) The device also reduced the net rate of metabolic energy expenditure for walking with an immobilized ankle from 23% above normal to 14%. Asterisks (*) denote statistical significance (*P*<0.01, paired *t*-tests, *n* = 11). Error bars denote s.d.

Normal walking yielded an average rate of ankle push-off work of 17.7±3.4 W (mean ± s.d., rate of positive work over a stride, Figure 3). The Conventional Prosthesis yielded lower values, at 9.8±1.4 W, similar to observations from amputee gait [1], [19], [20], [24]. The Energy Recycling foot captured energy from early in the stride at a rate of 6.9±0.7 W and returned it during push-off (Figure 3B). This energy capture resulted in substantially greater absorption than Normal at the ankle joint (11.0±3.4 W more), but little additional absorption for the entire leg and device during the same period (1.6±3.4 W). Recycling occurred with ground reaction forces similar to Normal (Figure S2). The recycled energy restored push-off to above Normal levels, at 18.9±1.5 W, about twice as much push-off as the Conventional Prosthesis (P = 1×10^−11^, paired t-test, Figure 4). Including the rest of the leg, push-off work was thus greater with the Energy Recycling foot than the Conventional Prosthesis, at 20.2±1.2 W vs. 14.3±2.0 W (P = 3×10^−8^).

The Conventional Prosthesis also increased metabolic energy expenditure, an energetic penalty that was reduced with the Energy Recycling foot (Figure 4). Normal walking yielded a net metabolic rate of 255±25 W above the rate of 125±23 W for quiet standing. Subjects expended 59±29 W more metabolic power than Normal with the Conventional Prosthetic foot, similar to differences observed between amputee and intact populations [2], [5]–[7]. Subjects expended only 35±21 W more than Normal with the Energy Recycling foot. The net metabolic penalty of walking with an immobilized ankle was therefore reduced from 23% with the Conventional foot to 14% using Energy Recycling (P = 3×10^−5^).

This reduction in metabolic energy expenditure compares favorably against a variety of conventional elastic prostheses, which have been found not to significantly reduce the metabolic penalty [5]–[7], and against other interventions for ankle impairment [2]. These savings appear to be associated with a reduction in the positive work performed by the human leg. We estimated the human contribution as total positive work over a stride minus work performed by the prosthesis (Figure S3), and found an overall reduction of 5.9±3.0 W with Energy Recycling compared to the Conventional Prosthesis (P = 7×10^−5^). Although the artificial foot absorbed more energy during collision, it primarily supplanted negative work usually performed by the human leg. Meanwhile, the increase in push-off due to recycling of this energy apparently reduced mechanical work requirements overall. It therefore appears that controlled storage and return of biomechanical energy provided a substantial metabolic benefit to walking with an immobilized ankle.

The precise relationship between push-off work and metabolic energy expenditure, however, is more complex than these results first imply. With the Conventional Prosthesis, ankle push-off decreased by 45% and net metabolic expenditure increased by 23% compared to Normal. The Energy Recycling foot restored push-off to 7% above the Normal level, but only reduced net metabolic energy expenditure by 9%. Some of the residual penalty may be due imprecise capture of energy, which appears to have caused additional positive work by the human leg during early stance (Figure S3). Some may be due to the relatively late timing of push-off in the Energy Recycling foot, which is theoretically less advantageous [15], [16]. Other issues could have contributed to this residual cost, such as the added mass of the simulator boot and device [21], [25], suboptimal curvature of the prosthesis or lift shoe bottom [21], or additional costs for swinging the legs [26]. These factors may be implicated by altered joint mechanics, such as at the hip and knee during swing (Figure S4 and Figure S5). Complicating interpretations further, it has even been observed that in some cases ankle push-off can be eliminated without causing a metabolic penalty [27]. In the present study, reduced push-off work appears to account for some, but not all, of the increased metabolic cost for walking with an impaired ankle.

This energy-recycling device may nevertheless provide a basis for the design of prosthetic feet that improve walking economy for amputees. The design would benefit from a reduction in weight and size, tuning of shape and stiffness characteristics for amputee gait, and improved cosmesis. Potential complexities due to the interface between residual limb and prosthesis would need to be studied.

Our results also suggest ways to improve other assistive devices. Energy recycling could be applied to other prosthetic limbs and orthotic devices, using configurations in parallel with the leg joints in addition to the series configuration examined here. Parallel devices would have the added advantage of reducing costs associated with force production [28]. Another possible energy source is negative work performed by the knee at the end of the swing phase, which might be mechanically recycled to aid leg motion [15], [29], or harvested by a generator to power other devices [30]. An alternative to recycling energy is simply to reduce the dissipation of the collision, as appears to be the effect of a backpack that reduces the energetic penalty of carrying an added load by supporting it on springs [31], [32]. Regardless of how energy is saved, an understanding of the negative work in walking may aid the design of powered human augmentation devices [8]–[10] and walking robots [33]. Devices based on these principles may even enable individuals with disabilities to outperform their able-bodied counterparts, allowing them to go further and faster with less effort.

## Supporting Information

Text S1 Additional descriptions of the artificial foot construction, experimental methods, and analysis methods. Includes supporting figures and captions.
(2.92 MB DOC)

Figure S1 Experimental setup. (A) Prosthesis simulator boots worn by intact subjects, fitted with the Energy Recycling foot or with the Conventional Prosthesis. Simulator boots were worn unilaterally (on the Affected leg), with a height-matched lift shoe on the opposite foot (Contralateral leg). Prosthesis simulator boots were comprised of AirCast© pneumatic boots augmented with a prosthetic pyramidal adaptor [21], [22]. (B) Mechanical and metabolic energy data were collected simultaneously using an instrumented split-belt treadmill [23] while subjects walked at 1.25 m s−1. A camera system and reflective markers were used to measure body and device motions, while force plates were used to measure ground reaction forces separately for each leg. Additionally, potentiometers measured prosthesis toe and heel rotations. Metabolic energy expenditure was estimated using indirect calorimetry.
(0.43 MB TIF)

Figure S2 Ground reaction forces. Normalized to body weight (BW, 82.6±7.1 N) and presented in components: (A) vertical component of the ground reaction force acting on the subject, with positive defined as opposing gravity, (B) fore-aft component with positive defined as along the direction of travel, and (C) lateral component with positive defined as rightward. Solid lines correspond to the leg on which the prosthesis simulator was worn (Affected leg), dashed lines correspond to the opposite limb (Contralateral leg). The stride begins at heel strike of the Affected limb. The first peak in vertical ground reaction force on the Contralateral limb was reduced with the Energy Recycling artificial foot as compared to the Conventional Prosthesis, apparently due to increased push-off impulse.
(0.48 MB TIF)

Figure S3 Center of mass work decomposition. Work performed on the center of mass over four phases of the gait cycle by the entire leg and by the human leg (un-shaded bars, estimated by subtracting separately-measured prosthesis work) for (A) the Affected leg (on which the prosthesis simulator was worn) and (B) the Contralateral leg. Collision, rebound, preload, and push-off refer to four characteristic phases of positive or negative center of mass work [11], [12], [18] (inset, cf. Figure 1C and cf. Figure 3). Work rate is defined as the sum of positive or negative work during each phase divided by the stride period. The contribution of each device was separately measured using inverse dynamics [20] and subtracted from center of mass work to estimate the work performed by the human leg during each phase. This estimate of human leg work can be visualized as the difference between the top and bottom panels of cf. Figure 3, calculated for each trial and averaged. Total Affected-limb push-off work was 42% greater with Energy Recycling than with the Conventional Prosthesis. Contralateral collision losses were 17% greater with the Conventional Prosthesis, despite shorter stride lengths in the Contralateral condition. Contralateral rebound work was 58% greater with the Conventional Prosthesis, presumably to balance the reduced push-off and increased collision. The sum of all positive center-of-mass work by both human legs over the course of a stride was 35.4±4.6 W with Energy Recycling and 41.4±3.3 W with the Conventional Prosthesis. This seems to account for the observed differences in metabolic cost between the conditions. Statistical significance between total work rates are shown in black while significance between human leg estimates are in gray. Error bars denote s.d., asterisks denote statistical significance at a level of P<0.01, and statistical comparisons of non-sequential conditions are not shown.
(0.54 MB TIF)

Figure S4 Lower-limb joint mechanics. Joint angles (top row), joint torques (middle row), and joint powers (bottom row) for the biological ankle (left column), knee (middle column), and hip (right column) as calculated using inverse dynamics [34,35]. Clinical phases of joint work [36] for the Affected side are marked as A1, A2, etc., as defined in the analysis methods section of Text S1. Solid lines correspond to the leg on which the prosthesis simulator was worn (Affected leg), dashed lines correspond to the opposite limb (Contralateral leg). The stride begins at heel strike of the Affected limb. In the Affected limb, the biological ankle joint was fixed in the prosthesis simulator, resulting in only minor displacement and work (not shown).
(0.57 MB TIF)

Figure S5 Lower limb joint work decomposition. Joint work was decomposed into clinical phases for (A) the Affected leg (on which the prosthesis simulator was worn) and (B) the Contralateral leg. Clinical phases of gait for each leg are defined in Figure S4 and in the analysis methods section of Text S1. Work rate is defined as the sum of positive or negative work during each phase divided by the stride period. Affected-limb H3 was 110% greater with the Conventional Prosthesis than with the Energy Recycling foot. K3 and K4 also increased significantly, possibly due to faster leg swing. A similar effect was observed in Contralateral-limb H3, K3, and K4. Conversely, Affected-limb H1 was 58% greater with the Energy Recycling foot, with the opposite effect in Contralateral H1, possibly an adaptation to enhance energy storage in the artificial foot during collision. Affected A1 and A2 data are unavailable because the ankle was immobilized by the prosthesis simulator in these conditions. Differences from Normal A2 in the Contralateral limb are an effect of the lift shoe. Error bars are standard deviation, asterisks denote statistical significance at a level of p<0.01, and statistical comparisons of non-sequential conditions are not shown.
(0.79 MB TIF)

Movie S1 Energy recycling with the artificial foot. High-speed video of the energy-recycling artificial foot, played back at 6% of actual speed. Camera rate was 500 frames per second. In the video, the foot proceeds through the phases described in Figure 2, beginning prior to heel strike and ending at reset. The foot is worn by an able-bodied individual using a below-knee prosthesis simulator boot. This demonstration was performed overground and with less-curved versions of the crepe roll-over shapes than used during testing.
(7.47 MB AVI)
