# Supplementary material for: Recycling Energy to Restore Impaired Ankle Function during Human Walking
Source: PLoS One. 2010 Feb 17;5(2):e9307. doi: 10.1371/journal.pone.0009307 (PMC2822861; doi:10.1371/journal.pone.0009307)
Supplement: Text S1 — Additional descriptions of the artificial foot construction, experimental methods, and analysis methods. Includes supporting figures and captions. (2.92 MB DOC) [file pone.0009307.s001.doc]

**Supporting Information**

Materials and Methods

*Description of Energy-Recycling Artificial Foot*

The energy-recycling artificial foot was comprised of six component groups: the attachment interface, the toe assembly, the heel assembly, the primary compression spring, the heel clutch, and the toe latch (cf. Figure 2). The interface component attached to the prosthesis simulator boot through a standard prosthetic pyramid adaptor (Figure S1). The one-way heel clutch acted between the heel assembly and the interface component, allowing the heel to rotate freely in plantar-flexion (compressing the spring) but locking when forced in the opposite direction (unless released by motor actuation). The toe latch acted between the toe assembly and the interface component, and prevented the toe assembly from rotating in plantar-flexion unless unlatched. During preload (about 25-40% stride), the torque acting on the toe assembly increased as the center of pressure advanced forward and ground reaction forces increased, eventually overcoming the torque of the primary compression spring and pushing the toe assembly against a limit stop. This relieved the toe latch of load, so that a motor could move it to effectively release the toe assembly. During push-off, the primary compression spring was then allowed to force the toe assembly through plantar-flexion, returning stored energy. Simultaneously, the carbon fiber toe spring returned energy that was stored during preload. At the onset of swing, the foot then reset into the ready position by unlocking the heel clutch (cf. Figure 2).

Prototype components were constructed of custom-machined 7075-T6 aluminum (interface component, toe and heel blocks), hardened O1 tool steel (latch surfaces), 416 stainless steel (shafts), and 0-90 carbon/fiberglass laminate (heel and toe leaf springs). The primary compression spring was a 2 inch long, 1.2 inch outer-diameter, chrome-vanadium steel die spring (9584K67; McMaster-Carr, Chicago, IL.). Two 10 mm coreless DC micro-motors with 64:1 gear reductions in planetary gear-heads (1016M012G+10/1K64:1; MicroMo, Clearwater, FL.) actuated the latches. Rotations of the toe and heel assemblies relative to the interface component were measured using contactless inductance-coil potentiometers (MP1545AS; P3 America Inc., San Diego, CA.). Sensory integration and control were performed by a Robostix microcontroller board (Gumstix, Inc., Portola Valley, CA.) based on an ATMega128 microcontroller chip (Atmel Co., San Jose, CA.).

*Experimental Methods*

The prosthesis simulator boot weighed 1.30 kg, and the lift shoe weighed 1.42 kg, with each adding approximately 0.129 m in leg length (Figure S1). In both the Energy Recycling and Conventional Prosthesis conditions, subjects wore a backpack containing a microcontroller which was connected to the simulator boot through a ribbon cable and connected to an analog data acquisition system through coaxial cables.

During training, subjects walked under each condition, including normal walking, for ten minutes. Subjects were given an initial acclimation period of five to ten minutes of self-selected overground walking with both the Energy Recycling foot and Conventional Prosthesis prior to collections.

For energetics calculations, we measured the rate of oxygen consumption ( in ml O_2_/sec) and carbon dioxide production ( in ml CO_2_/sec) using an open-circuit respirometry system (Physio-Dyne Instrument, Quogue, NY). Each trial lasted at least ten minutes, including at least six minutes to allow subjects to adapt and reach steady state, followed by three minutes of data recording for average and during steady state. Metabolic rates (in Watts) were estimated from oxygen and carbon-dioxide rates with the derived formula [34]

We also measured each subject’s metabolic rate for quiet standing in a separate trial and subtracted it from the rate for walking to yield net metabolic rate. All conditions, including quiet standing, were conducted in random order. Respiratory exchange ratios were found to be less than unity for all subjects and conditions, indicating that energy was supplied primarily by oxidative metabolism in all conditions.

For mechanics calculations, we measured kinematics and ground reaction forces as subjects walked on an instrumented treadmill. Kinematic data were recorded with an 8-camera motion capture system (Motion Analysis Corporation, Santa Rosa, CA) at 120 Hz. Force data were recorded at 1200 Hz using an instrumented split-belt treadmill [23]. Treadmill belt speed was maintained at 1.25 m s^-1^. We recorded at least 40 consecutive strides per condition for each subject. For inverse dynamics analysis, a set of motion capture markers were placed bilaterally on the lower extremities according to a modified Helen Hayes marker set. In conditions where subjects wore a prosthesis simulator boot, markers were placed on the simulator boot in locations approximating the same bony landmarks. In Conventional Prosthesis trials, markers were placed on the heel, fifth metatarsal, and lateral malleolli equivalents of the prosthesis, as is common practice [19]. During the Energy Recycling condition, markers were rigidly attached to the foot on both ends of the shaft, on the interface component, on the tip of the heel and on the tip of the toe.

*Analysis Methods*

We calculated joint power and work performed on the center of mass for all conditions. We estimated joint power using standard inverse dynamics analysis [35,36]. Distal link endpoint forces were measured using force plates during ground contact and were assumed to be zero during swing phases. Anthropometric data were estimated from the equations of Winter [35], and were augmented to include the mass properties of the prosthesis simulator and lift shoes, which were measured directly. Velocities and torques were low-pass filtered at 25 Hz. Joint rotations, torques, and powers were calculated within the sagittal plane. We estimated the work performed on the center of mass by each leg [12], based on the vector dot product of each leg’s ground reaction force against the center of mass velocity.

We calculated power for the energy-recycling foot and conventional prosthesis using inverse dynamics in the manner of Prince & Winter [20] which accounts for both translational and rotational modes of energy absorption and return [19]. Inertial properties used in this calculation were estimated using the component CAD models (Solidworks, Concord MA) and direct measurement. Prosthesis rotations were calculated using markers. Additionally, potentiometer data was used to measure compression spring motion in the energy-recycling artificial foot, which differed from whole-foot mechanics due to compliance and dissipation in the heel and toe keels.

Each stride was normalized to percent stride and averaged for each subject and condition. All quantities were analyzed in dimensionless form, to help account for variations in subject size. Torque and work quantities were normalized by each subject’s body weight and leg length (*MgL*, where *M* is body mass, *g* is gravitational acceleration, and *L* is leg length), with the additional factor of *g*^0.5^*L*^-0.5^ (the leg’s pendulum frequency) for work rate quantities. Averages, standard deviations, and statistics were computed in dimensionless quantities. We report variables in familiar dimensional units such as W kg^-1^, converted using average normalization factors. Average normalization factors were: 873 kg·m^2^·s^-2^ for torque and mechanical work, 2.63·10^3^ kg·m^2^·s^-3^ for work rates and metabolic rate, and 810 N for forces.

We compared average work rates for the different conditions in separate phases, as follows. The center of mass work performed by the entire leg and device was divided into collision, rebound, preload, and push-off (Figure S3) with boundaries determined by successive zero-crossings of the center of mass work rate (these can be seen clearly in cf. Figures 1 and 3). Joint work was divided into nine phases used in clinical gait analysis [37] (Figures S4 & S5): Ankle work was divided into an A1 phase beginning at initial contact and ending at the zero-crossing at the onset of ankle push-off, and an A2 phase comprised of ankle push-off. Knee work was divided into a K1 phase beginning at initial contact and ending at the zero-crossing near the end of double support, a K2 phase ending at the zero-crossing at the end of single-support, a K3 phase ending at the zero-crossing of knee torque during mid-swing, and a K4 phase comprised of terminal swing. Hip work was divided into an H1 phase beginning at initial contact and ending at the zero-crossing near the end of double-support, an H2 phase ending at the zero-crossing during the subsequent double-support, and an H3 phase comprising the end of double-support and swing.

Each phase of joint and center of mass work was considered in terms of work rate, with positive and negative contributions considered separately. Work rate was defined as the total positive or negative work performed during the phase divided by the stride time. Thus, contributions of each phase of center of mass or joint work rate can be compared across conditions with differing stride frequency.

We statistically compared outcome variables that captured the primary energetics and mechanics results. We compared net metabolic rate, work rate during leg work phases, work rate during joint phases, and work rates of the artificial foot and prosthetic foot. Statistical comparisons were made with repeated measures ANOVA for each variable, with a significance level of 0.05. Where differences were significant, post hoc comparisons were performed using paired t-tests according to the Sidak-Holm step-down procedure.

Figures

**Figure S1. Experimental setup.** (**A**) Prosthesis simulator boots worn by intact subjects, fitted with the Energy Recycling foot or with the Conventional Prosthesis. Simulator boots were worn unilaterally (on the Affected leg), with a height-matched lift shoe on the opposite foot (Contralateral leg). Prosthesis simulator boots were comprised of AirCast© pneumatic boots augmented with a prosthetic pyramidal adaptor [21,22]. (**B**) Mechanical and metabolic energy data were collected simultaneously using an instrumented split-belt treadmill [23] while subjects walked at 1.25 m s^-1^. A camera system and reflective markers were used to measure body and device motions, while force plates were used to measure ground reaction forces separately for each leg. Additionally, potentiometers measured prosthesis toe and heel rotations. Metabolic energy expenditure was estimated using indirect calorimetry.

**Figure S2. Ground reaction forces.** Normalized to body weight (BW, 810 ± 70 N) and presented in components: (**A**) vertical component of the ground reaction force acting on the subject, with positive defined as opposing gravity, (**B**) fore-aft component with positive defined as along the direction of travel, and (**C**) lateral component with positive defined as rightward. Solid lines correspond to the leg on which the prosthesis simulator was worn (Affected leg), dashed lines correspond to the opposite limb (Contralateral leg). The stride begins at heel strike of the Affected limb. The first peak in vertical ground reaction force on the Contralateral limb was reduced with the Energy Recycling artificial foot as compared to the Conventional Prosthesis, apparently due to increased push-off impulse.

**Figure S3**. **Center of mass work decomposition.** Work performed on the center of mass over four phases of the gait cycle by the entire leg and by the human leg (un-shaded bars, estimated by subtracting separately-measured prosthesis work) for (**A**) the Affected leg (on which the prosthesis simulator was worn) and (**B**) the Contralateral leg. Collision, rebound, preload, and push-off refer to four characteristic phases of positive or negative center of mass work [11,12,18] (inset, cf. Figure 1C and cf. Figure 3). Work rate is defined as the sum of positive or negative work during each phase divided by the stride period. The contribution of each device was separately measured using inverse dynamics [20] and subtracted from center of mass work to estimate the work performed by the human leg during each phase. This estimate of human leg work can be visualized as the difference between the top and bottom panels of cf. Figure 3, calculated for each trial and averaged. Total Affected-limb push-off work was 42% greater with Energy Recycling than with the Conventional Prosthesis. Contralateral collision losses were 17% greater with the Conventional Prosthesis, despite shorter stride lengths in the Contralateral condition. Contralateral rebound work was 58% greater with the Conventional Prosthesis, presumably to balance the reduced push-off and increased collision. The sum of all positive center-of-mass work by both human legs over the course of a stride was 35.4 ± 4.6 W with Energy Recycling and 41.4 ± 3.3 W with the Conventional Prosthesis. This seems to account for the observed differences in metabolic cost between the conditions. Statistical significance between total work rates are shown in black while significance between human leg estimates are in gray. Error bars denote s.d., asterisks denote statistical significance at a level of *P* < 0.01, and statistical comparisons of non-sequential conditions are not shown.

**Figure S4.** **Lower-limb joint mechanics.** Joint angles (top row), joint torques (middle row), and joint powers (bottom row) for the biological ankle (left column), knee (middle column), and hip (right column) as calculated using inverse dynamics [35,36]. Clinical phases of joint work [37] for the Affected side are marked as A1, A2, etc., as defined in the analysis methods section of the supporting text. Solid lines correspond to the leg on which the prosthesis simulator was worn (Affected leg), dashed lines correspond to the opposite limb (Contralateral leg). The stride begins at heel strike of the Affected limb. In the Affected limb, the biological ankle joint was fixed in the prosthesis simulator, resulting in only minor displacement and work (not shown).

**Figure S5**. **Lower limb joint work decomposition.** Joint work was decomposed into clinical phases for (**A**) the Affected leg (on which the prosthesis simulator was worn) and (**B**) the Contralateral leg. Clinical phases of gait for each leg are defined in Figure S4 and in the analysis methods section of the supporting text. Work rate is defined as the sum of positive or negative work during each phase divided by the stride period. Affected-limb H3 was 110% greater with the Conventional Prosthesis than with the Energy Recycling foot. K3 and K4 also increased significantly, possibly due to faster leg swing. A similar effect was observed in Contralateral-limb H3, K3, and K4. Conversely, Affected-limb H1 was 58% greater with the Energy Recycling foot, with the opposite effect in Contralateral H1, possibly an adaptation to enhance energy storage in the artificial foot during collision. Affected A1 and A2 data are unavailable because the ankle was immobilized by the prosthesis simulator in these conditions. Differences from Normal A2 in the Contralateral limb are an effect of the lift shoe. Error bars are standard deviation, asterisks denote statistical significance at a level of p < 0.01, and statistical comparisons of non-sequential conditions are not shown.

SOM Movie Caption

**Movie S1. Energy recycling with the artificial foot.** High-speed video of the energy-recycling artificial foot, played back at 6% of actual speed. Camera rate was 500 frames per second. In the video, the foot proceeds through the phases described in Figure 2, beginning prior to heel strike and ending at reset. The foot is worn by an able-bodied individual using a below-knee prosthesis simulator boot. This demonstration was performed overground and with less-curved versions of the crepe roll-over shapes than used during testing.

References

34. Brockway JM (1987) Derivation of formulae used to calculate energy expenditure in man. Hum Nutr Clin Nutr 41: 463-471.

35. Winter DA (1990) Biomechanics and Motor Control of Human Movement. Toronto: John Wiley & Sons, Inc.

36. Siegler S, Liu W (1998) Inverse dynamics in human locomotion. In: Allard P, Cappozzo A, Lundberg A, Vaughan CL, editors. Three-Dimensional Analysis of Human Locomotion. New York: John Wiley & Sons. pp. 191-209.

37. Whittle M (1996) Gait Analysis: An Introduction. Oxford: Butterworth-Heinemann Medical.
