# Supplementary figures and images for: Recycling Energy to Restore Impaired Ankle Function during Human Walking
Source: PLoS One. 2010 Feb 17;5(2):e9307. doi: 10.1371/journal.pone.0009307 (PMC2822861; doi:10.1371/journal.pone.0009307)

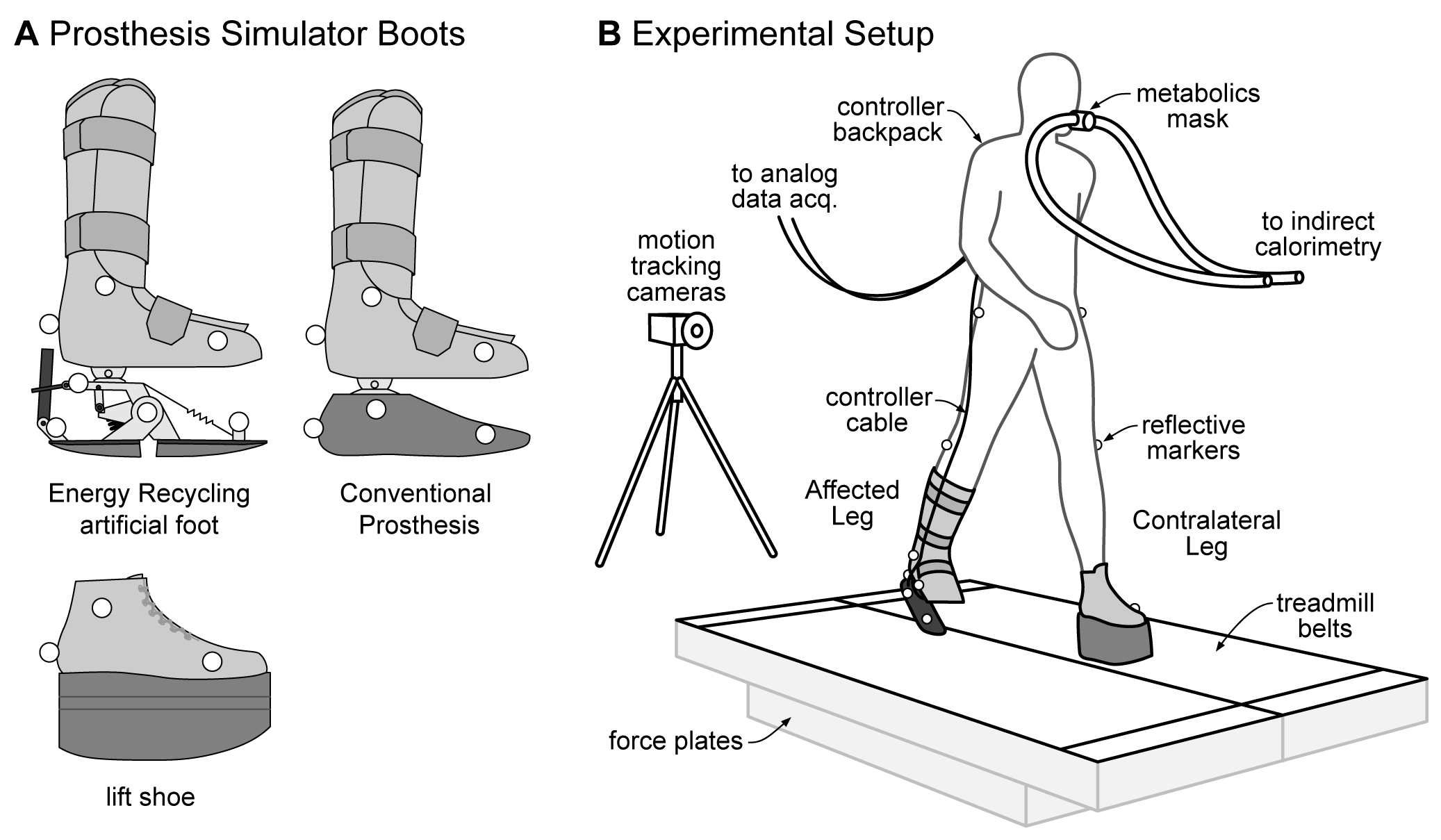

Supplement: Figure S1 — Experimental setup. (A) Prosthesis simulator boots worn by intact subjects, fitted with the Energy Recycling foot or with the Conventional Prosthesis. Simulator boots were worn unilaterally (on the Affected leg), with a height-matched lift shoe on the opposite foot (Contralateral leg). Prosthesis simulator boots were comprised of AirCast© pneumatic boots augmented with a prosthetic pyramidal adaptor [21], [22]. (B) Mechanical and metabolic energy data were collected simultaneously using an instrumented split-belt treadmill [23] while subjects walked at 1.25 m s−1. A camera system and reflective markers were used to measure body and device motions, while force plates were used to measure ground reaction forces separately for each leg. Additionally, potentiometers measured prosthesis toe and heel rotations. Metabolic energy expenditure was estimated using indirect calorimetry. (0.43 MB TIF) [file pone.0009307.s002.tif]

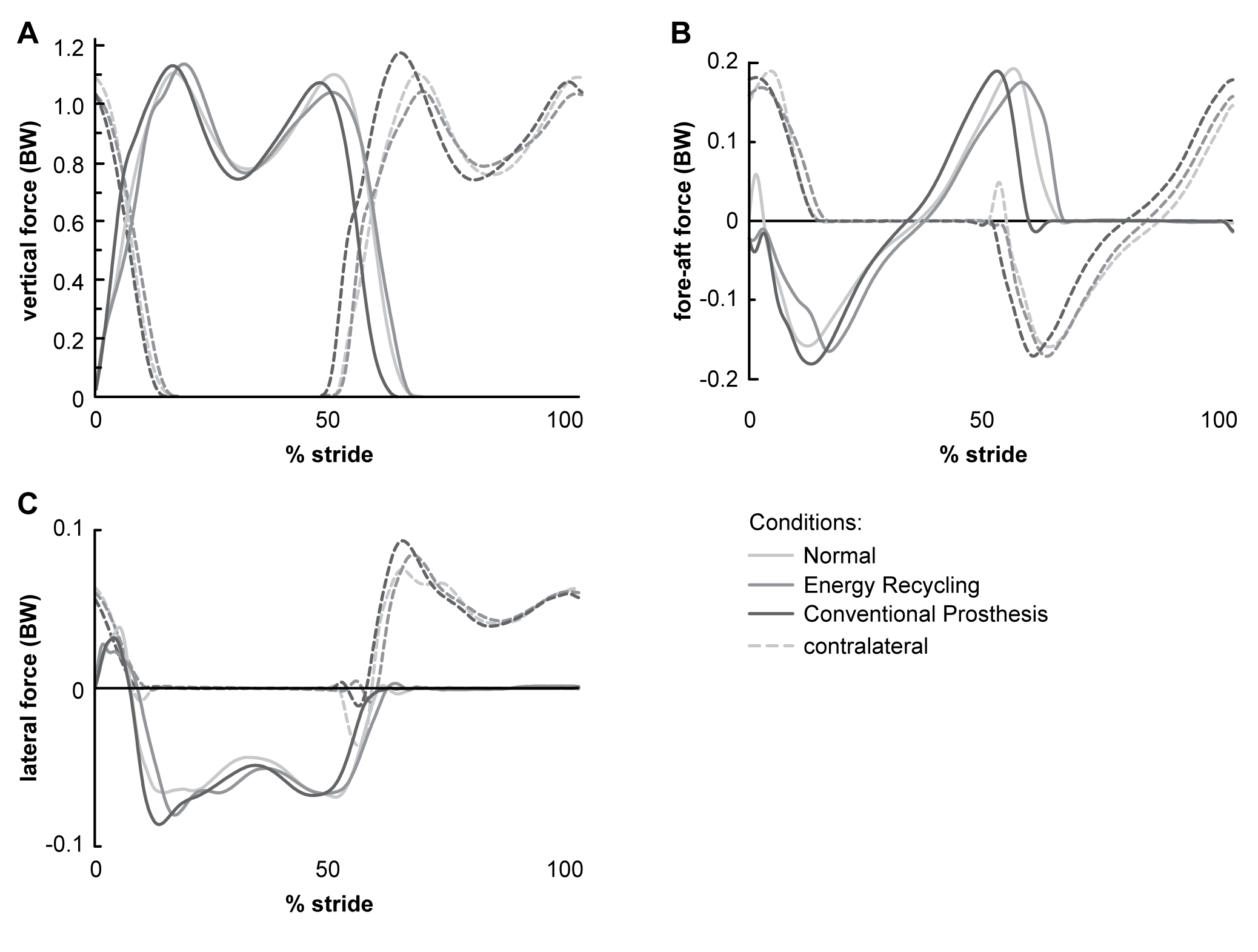

Supplement: Figure S2 — Ground reaction forces. Normalized to body weight (BW, 82.6±7.1 N) and presented in components: (A) vertical component of the ground reaction force acting on the subject, with positive defined as opposing gravity, (B) fore-aft component with positive defined as along the direction of travel, and (C) lateral component with positive defined as rightward. Solid lines correspond to the leg on which the prosthesis simulator was worn (Affected leg), dashed lines correspond to the opposite limb (Contralateral leg). The stride begins at heel strike of the Affected limb. The first peak in vertical ground reaction force on the Contralateral limb was reduced with the Energy Recycling artificial foot as compared to the Conventional Prosthesis, apparently due to increased push-off impulse. (0.48 MB TIF) [file pone.0009307.s003.tif]

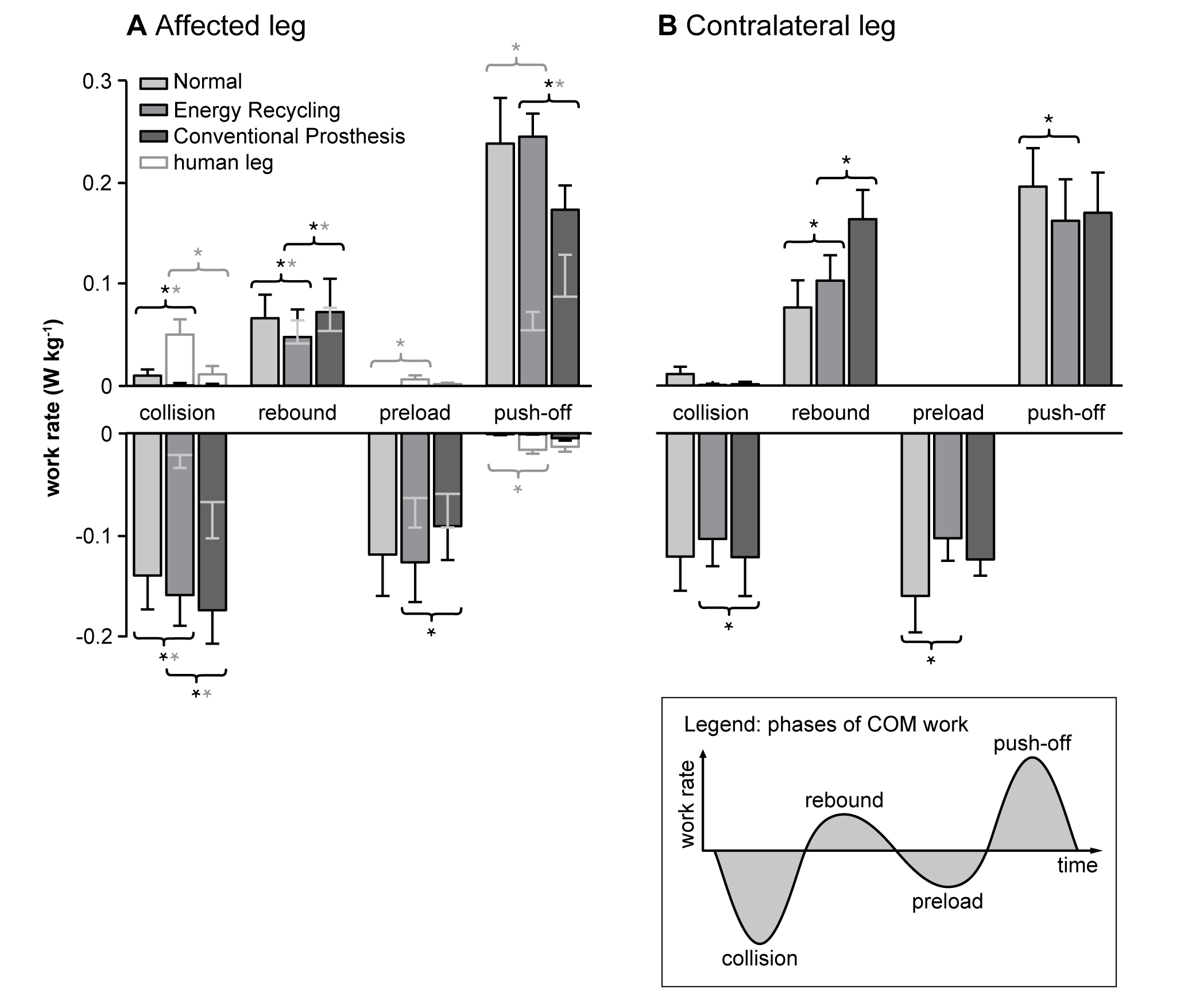

Supplement: Figure S3 — Center of mass work decomposition. Work performed on the center of mass over four phases of the gait cycle by the entire leg and by the human leg (un-shaded bars, estimated by subtracting separately-measured prosthesis work) for (A) the Affected leg (on which the prosthesis simulator was worn) and (B) the Contralateral leg. Collision, rebound, preload, and push-off refer to four characteristic phases of positive or negative center of mass work [11], [12], [18] (inset, cf. Figure 1C and cf. Figure 3). Work rate is defined as the sum of positive or negative work during each phase divided by the stride period. The contribution of each device was separately measured using inverse dynamics [20] and subtracted from center of mass work to estimate the work performed by the human leg during each phase. This estimate of human leg work can be visualized as the difference between the top and bottom panels of cf. Figure 3, calculated for each trial and averaged. Total Affected-limb push-off work was 42% greater with Energy Recycling than with the Conventional Prosthesis. Contralateral collision losses were 17% greater with the Conventional Prosthesis, despite shorter stride lengths in the Contralateral condition. Contralateral rebound work was 58% greater with the Conventional Prosthesis, presumably to balance the reduced push-off and increased collision. The sum of all positive center-of-mass work by both human legs over the course of a stride was 35.4±4.6 W with Energy Recycling and 41.4±3.3 W with the Conventional Prosthesis. This seems to account for the observed differences in metabolic cost between the conditions. Statistical significance between total work rates are shown in black while significance between human leg estimates are in gray. Error bars denote s.d., asterisks denote statistical significance at a level of P<0.01, and statistical comparisons of non-sequential conditions are not shown. (0.54 MB TIF) [file pone.0009307.s004.tif]

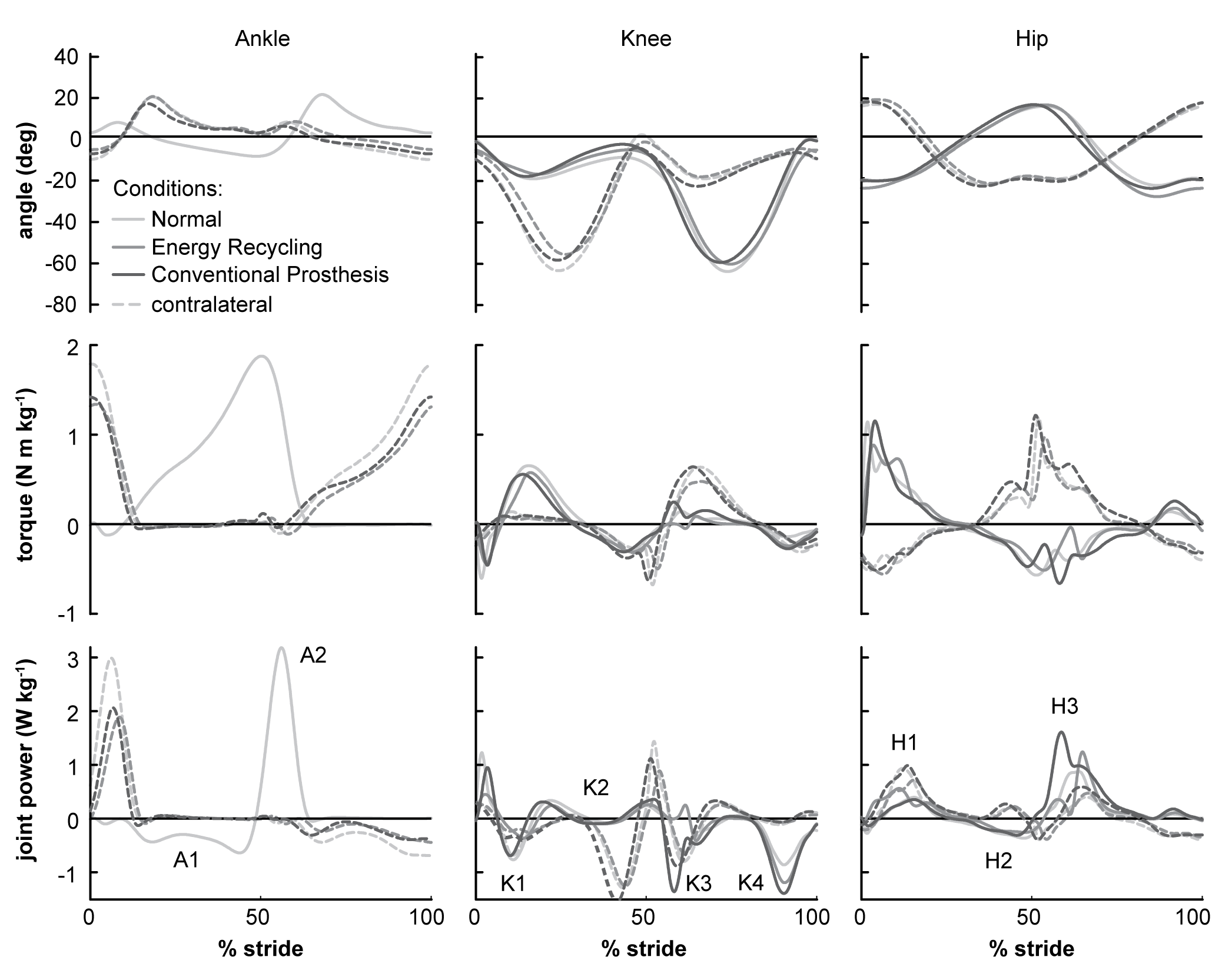

Supplement: Figure S4 — Lower-limb joint mechanics. Joint angles (top row), joint torques (middle row), and joint powers (bottom row) for the biological ankle (left column), knee (middle column), and hip (right column) as calculated using inverse dynamics [34,35]. Clinical phases of joint work [36] for the Affected side are marked as A1, A2, etc., as defined in the analysis methods section of Text S1. Solid lines correspond to the leg on which the prosthesis simulator was worn (Affected leg), dashed lines correspond to the opposite limb (Contralateral leg). The stride begins at heel strike of the Affected limb. In the Affected limb, the biological ankle joint was fixed in the prosthesis simulator, resulting in only minor displacement and work (not shown). (0.57 MB TIF) [file pone.0009307.s005.tif]

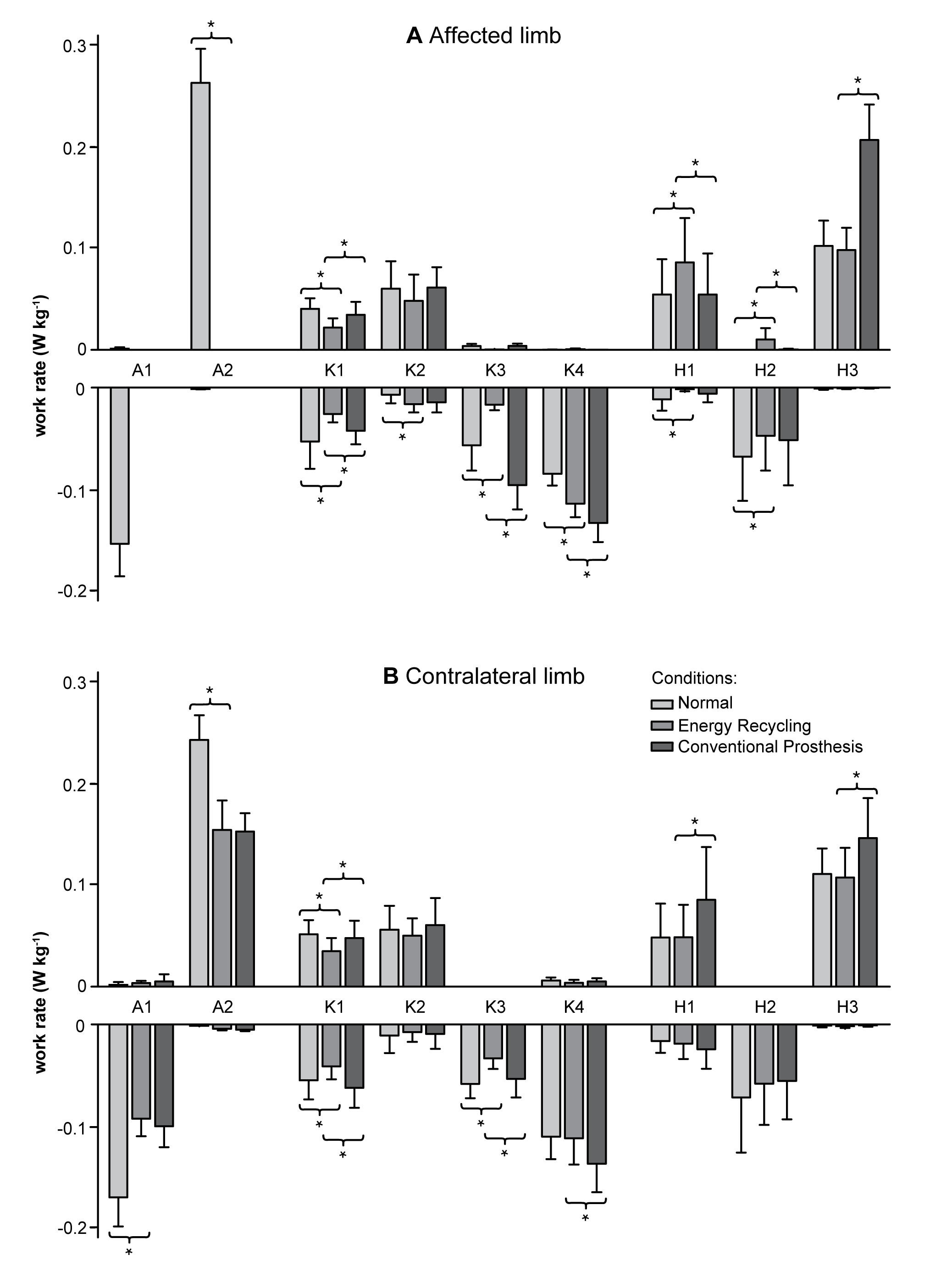

Supplement: Figure S5 — Lower limb joint work decomposition. Joint work was decomposed into clinical phases for (A) the Affected leg (on which the prosthesis simulator was worn) and (B) the Contralateral leg. Clinical phases of gait for each leg are defined in Figure S4 and in the analysis methods section of Text S1. Work rate is defined as the sum of positive or negative work during each phase divided by the stride period. Affected-limb H3 was 110% greater with the Conventional Prosthesis than with the Energy Recycling foot. K3 and K4 also increased significantly, possibly due to faster leg swing. A similar effect was observed in Contralateral-limb H3, K3, and K4. Conversely, Affected-limb H1 was 58% greater with the Energy Recycling foot, with the opposite effect in Contralateral H1, possibly an adaptation to enhance energy storage in the artificial foot during collision. Affected A1 and A2 data are unavailable because the ankle was immobilized by the prosthesis simulator in these conditions. Differences from Normal A2 in the Contralateral limb are an effect of the lift shoe. Error bars are standard deviation, asterisks denote statistical significance at a level of p<0.01, and statistical comparisons of non-sequential conditions are not shown. (0.79 MB TIF) [file pone.0009307.s006.tif]
